# Safety Concerns, Mechanistic Pathways, and Knowledge Gaps in the Clinical Use of Selective Serotonin Reuptake Inhibitors

**DOI:** 10.7759/cureus.100719

**Published:** 2026-01-03

**Authors:** Adrian Chin Yan Chan

**Affiliations:** 1 Benefit-Risk Management, Bayers Pharmaceuticals, Shanghai, CHN

**Keywords:** discontinuation syndrome, drug interactions, pharmacovigilance, psychiatric disorders, regulatory perspectives, safety concerns, serotonin syndrome, ssris

## Abstract

Selective serotonin reuptake inhibitors (SSRIs) are widely prescribed for the management of depressive and anxiety disorders and represent one of the most significant pharmacological advances in psychiatry. SSRIs are effective and better tolerated than older antidepressants; however, long-term safety issues, interactions, and withdrawal effects remain a concern. This review combines data from clinical trials, safety databases, and biological studies to provide a clear overview of the safety of SSRIs. The key risks include gastrointestinal bleeding, sexual dysfunction, hyponatremia, serotonin syndrome, discontinuation syndromes, and cardiovascular complications. Recent studies have reported lesser-known problems, such as akathisia, post-SSRI sexual dysfunction, metabolic issues, and possible effects on cognition. Large-scale pharmacovigilance analyses, such as those of the WHO VigiBase and Food and Drug Administration Adverse Event Reporting System, demonstrate that certain SSRIs carry disproportionate reporting signals, underscoring the need for individualized prescribing and long-term monitoring. This review further discusses the genetic and pharmacogenomic determinants of treatment response, as well as special population risks, such as those in the elderly, adolescents, and pregnant women, and regulatory measures aimed at mitigating harm. By critically integrating clinical, mechanistic, and real-world evidence, this review identifies significant knowledge gaps, particularly concerning long-term safety and understudied populations, and offers recommendations for future research. Clinicians should adopt a patient-centered risk-benefit evaluation, balancing therapeutic efficacy with safety concerns.

## Introduction and background

Historical development and mechanistic foundation

Selective serotonin reuptake inhibitors (SSRIs) emerged in the late 20th century as a major advancement in managing depression and other mental health conditions, developed specifically as safer alternatives to tricyclic antidepressants (TCAs) and monoamine oxidase inhibitors (MAOIs). The introduction of fluoxetine (Prozac) in 1987 marked a turning point in psychopharmacology, as its improved tolerability profile compared to older agents accelerated widespread clinical adoption [1,2]. Prior to SSRIs, TCAs, and MAOIs dominated depression pharmacotherapy but carried significant anticholinergic, cardiovascular, and dietary interaction risks that necessitated the development of more selective and tolerable agents [3].

The mechanistic rationale underlying SSRI development stemmed from the serotonergic hypothesis, which posits that reduced serotonergic neurotransmission contributes to depressive symptoms. To enhance accessibility for non-specialist readers, it is important to note that serotonergic signaling involves the release of serotonin into the synaptic cleft, where it binds to postsynaptic receptors; SSRIs block the reuptake of serotonin by inhibiting the serotonin transporter (SERT), thereby increasing synaptic serotonin availability and modulating mood, anxiety, and related neurophysiological processes. This hypothesis guided early SSRI development and continues to provide an organizing framework for understanding both therapeutic effects and certain adverse reactions (particularly those related to serotonergic excess, such as serotonin syndrome) [3-5]. However, contemporary neuroscience emphasizes that depression is multifactorial, and simplistic monoamine models inadequately account for clinical heterogeneity, variable treatment response, and the diverse safety profile of SSRIs observed in real-world practice.

Clinical indications and expanding use

Following fluoxetine, several SSRIs (e.g., sertraline, citalopram, and escitalopram) were introduced and are now established as first-line pharmacotherapies for major depressive disorder (MDD) and many anxiety disorders (generalized anxiety disorder, panic disorder, obsessive-compulsive disorder (OCD), and post-traumatic stress disorder) [6]. The therapeutic versatility of SSRIs has led to expanding use beyond regulatory-approved psychiatric indications into off-label applications such as premenstrual dysphoric disorder (PMDD), select chronic pain conditions (e.g., fibromyalgia), and premature ejaculation, reflecting their broad prescribing across both psychiatric and non-psychiatric clinical settings [7,8].

While SSRIs demonstrate superior tolerability compared to older antidepressants, a characteristic that has facilitated their rapid clinical uptake, this relative safety advantage does not eliminate the need for systematic safety evaluation [2]. SSRIs remain associated with clinically important risks, including serotonin syndrome, treatment-emergent suicidal ideation (particularly in younger patients), gastrointestinal (GI) bleeding (especially when co-prescribed with NSAIDs or anticoagulants), sexual dysfunction (both during and potentially persisting after treatment discontinuation, including post-SSRI sexual dysfunction (PSSD)), and discontinuation/withdrawal syndromes following abrupt cessation [9-11]. The clinical significance of these safety considerations has intensified in parallel with global trends in long-term SSRI use and increasing prescription volumes across diverse patient populations.

Global usage patterns: epidemiological context for safety surveillance

The prescription and consumption of SSRIs (and other antidepressants) have increased markedly over the past three decades, especially in high-income countries. Per-capita antidepressant consumption (measured as defined daily doses per 1000 inhabitants) is substantially higher in wealthier nations than in low-income countries, a pattern that reflects differences in access to mental health services, diagnostic capacity, and prescribing practices [9]. International DDD analyses and OECD reports document these intercountry disparities [12,13]. Recent national data also show sustained increases in outpatient antidepressant dispensing since 2020 in many settings, with notable increases among adolescents and young adults in the US and Europe [14,15].

These global usage patterns carry critical implications for pharmacovigilance and safety monitoring. Higher population-level SSRI exposure in resource-rich settings translates directly to increased absolute numbers of patients experiencing adverse drug reactions, even if individual-level risk remains constant, a phenomenon that amplifies the public health burden of both common (e.g., sexual dysfunction and GI symptoms) and rare but serious adverse events (e.g., serotonin syndrome, syndrome of inappropriate antidiuretic hormone secretion (SIADH)-associated hyponatremia, and cardiac conduction abnormalities). Conversely, in low-use settings with limited pharmacovigilance infrastructure, opportunities for early detection of safety signals may be missed, potentially delaying regulatory responses and clinical guideline updates. The combination of rising long-term SSRI use in high-income countries and persistent undertreatment of mental illness in resource-limited settings creates a dual public health challenge: in high-use populations, there exists an urgent need for ongoing post-marketing surveillance and prescribing stewardship to minimize preventable harm, while globally, mental health conditions remain substantially underdiagnosed and undertreated [9,13,15].

Clinical importance and scope

Within this epidemiological context, SSRIs maintain their position as front-line pharmacologic agents for MDD, justified by their favorable balance of efficacy and tolerability across many patient subgroups [6]. Large-scale umbrella reviews and contemporary meta-analyses confirm that antidepressants as a therapeutic class demonstrate superior efficacy relative to placebo for depressive disorders; however, observed effect sizes show considerable heterogeneity, and clinical trial data consistently document increased rates of adverse events and treatment discontinuation in active treatment arms compared to placebo [8]. These findings underscore the necessity of individualized risk-benefit evaluation rather than population-averaged treatment recommendations. SSRIs also demonstrate established efficacy across anxiety disorder spectrum conditions and OCD, and in specific patient populations, they reduce relapse risk during maintenance therapy phases [4,7].

Rationale for review

Despite widespread prescribing and generally favorable tolerability relative to older antidepressants, SSRIs present complex and sometimes under-recognized safety challenges that demand systematic, evidence-based evaluation integrating multiple data sources. Existing literature reviews often emphasize short-term efficacy outcomes while underemphasizing safety considerations, creating knowledge gaps in several critical domains: 1) Incomplete adverse event reporting in clinical trials: randomized controlled trials systematically underreport adverse events due to short follow-up periods, selected patient populations (exclusion of comorbidities and polypharmacy), and variable adverse event definitions that limit comparability across studies [16,17]. 2) Limited integration of complementary evidence sources: traditional efficacy-focused reviews rarely integrate findings from large-scale pharmacovigilance databases (WHO VigiBase, Food and Drug Administration (FDA) Adverse Event Reporting System (FAERS)) with mechanistic research and clinical trial data, despite these sources offering complementary insights into rare, delayed, and population-specific adverse events [18-20]. 3) Emerging evidence of persistent or late-onset harm: growing recognition of potentially persistent adverse effects, including PSSD and protracted withdrawal syndromes that may persist for months or years, challenges assumptions that adverse events invariably resolve upon drug discontinuation [16,17]. 4) Population-specific safety profiles are insufficiently characterized: special populations, including pregnant women, adolescents, elderly patients, and those with medical comorbidities, remain underrepresented in safety research despite distinct physiological vulnerabilities and altered risk-benefit profiles.

This review, therefore, adopts a deliberately safety-centered approach, systematically synthesizing evidence from randomized controlled trials, observational pharmacoepidemiology, global pharmacovigilance databases, and mechanistic neurobiological research. Unlike the background sections above, which establish the historical development, mechanistic basis, clinical indications, and epidemiological context of SSRI use, this review focuses specifically on under-recognized long-term adverse effects, clinically significant drug-drug interactions, age-specific and population-specific safety considerations, and their implications for evidence-based prescribing policy, clinical monitoring protocols, and patient counseling strategies.

The main goal is to provide clinicians, patients, and health policymakers with a comprehensive, balanced appraisal of SSRI safety that facilitates optimization of therapeutic benefits while proactively minimizing avoidable harm through informed clinical decision-making and appropriate monitoring.

Search and selection methodology

This narrative review was informed by a structured literature search conducted in PubMed, Embase, Scopus, and Cochrane Library. Search terms included combinations of “Selective Serotonin Reuptake Inhibitors,” “SSRI safety,” “adverse effects,” “pharmacovigilance,” and “long-term outcomes,” covering publications from 1990 to December 2024. Additional regulatory documents were screened from the FDA, EMA, and WHO databases. Inclusion criteria were: peer-reviewed human studies, systematic reviews, meta-analyses, pharmacovigilance data, clinical trials, and major regulatory communications relevant to SSRI safety. Exclusion criteria included non-English articles, preclinical studies unless directly linked to mechanistic insights, and duplicate data. As this is a narrative review, no formal risk-of-bias tool was applied, but study design, sample size, and methodological rigor were considered when interpreting findings. This review does not include a pooled statistical synthesis (e.g., meta-analysis) because it is designed as a narrative integration of clinical, mechanistic, and pharmacovigilance evidence. Where meta-analyses are cited, numerical outcomes such as effect sizes, odds ratios, and confidence intervals have been included to enhance analytical clarity. As no new statistical analysis was performed, additional statistical peer review is not required (Appendix A and Appendix B).

## Review

Mechanism of action of selective serotonin reuptake inhibitors

SSRI mechanisms span established pharmacology, confirmed through radioligand binding studies and clinical pharmacokinetics, and emerging hypotheses based on preclinical models and correlational human data. We distinguish these levels of evidence throughout this section, providing quantitative receptor-binding parameters where available to contextualize inter-agent variability and clinical consequences.

Serotonin Reuptake Inhibition

SSRIs are a class of drugs widely prescribed for the treatment of depressive disorders because of their favorable efficacy and tolerability, which support their use as first-line pharmacotherapy in many clinical guidelines [21,22]. Their canonical mechanism is the competitive inhibition of SERT on presynaptic terminals, reducing serotonin reuptake and thereby increasing extracellular serotonin concentrations in the synaptic cleft [23]. This process occurs rapidly at pharmacologic doses but does not explain the delayed clinical response [24].

Serotonin Transporter Involvement and Pharmacogenetics

SERT (encoded by SLC6A4) remains the primary molecular target for SSRIs, but therapeutic outcomes are influenced by interindividual variation in transporter expression and regulation [1,22]. Pharmacogenetic studies and meta-analyses support the role of polymorphisms in the SLC6A4 promoter region (5-HTTLPR) and related methylation patterns in moderating antidepressant response and risk of adverse effects, although effect sizes are modest and findings vary by ancestry and study design [25,26]. Quantitatively, the 5-HTTLPR short allele is associated with approximately 30%-40% lower SERT expression compared to the long allele, though clinical effect sizes for treatment response remain small (Cohen's d=0.1-0.2) and require validation across diverse populations [25]. Recent systematic analyses highlighted both sequence polymorphisms and epigenetic modifications (promoter methylation) as contributors to heterogeneous treatment outcomes, underscoring a multifactorial genetic architecture rather than a single predictive marker [27-29].

Intracellular and Subcellular Drug Dynamics

Beyond SERT blockade at the plasma membrane, contemporary pharmacokinetic and cell biology studies have demonstrated that SSRIs are distributed across cellular compartments (cytoplasm, endoplasmic reticulum (ER), and membranes) in a time-dependent manner. Subcellular partitioning may influence the duration of transporter occupancy and off-target receptor exposure. Experimental evidence from fluorescence microscopy demonstrates SSRI accumulation in the ER, with ER:cytoplasm concentration ratios of 3-10:1 for fluoxetine and paroxetine, though clinical correlates remain under investigation [26,30,31]. Two recent reviews with experimental data and theoretical integration, such as Nichols 2023 and Blumenfeld et al. 2023, emphasize that intracellular accumulation and organellar concentrations are underappreciated determinants of SSRI pharmacology and may help explain delayed adaptive responses [26,31].

Delayed Therapeutic Effects and Neuroplasticity

Although SERT occupancy occurs within hours of dosing, clinical antidepressant effects commonly appear after two to six weeks, a latency attributed to downstream neuroadaptive processes [30]. Mechanistic candidates supported by recent preclinical and clinical studies include enhanced hippocampal neurogenesis, upregulation of neurotrophic signaling pathways (e.g., brain-derived neurotrophic factor (BDNF)), synaptic remodeling, and neural circuit changes that alter emotional bias processing. These neuroplastic changes have been summarized in recent comprehensive reviews of antidepressant mechanisms, which stress the interactions among neurotransmission, neuroinflammation, and plasticity [24,32].

Off-Target Binding: Transporters, Serotonin Receptors, and Clinical Consequences

SSRIs are relatively selective for SERT but show low-affinity interactions with other monoamine transporters (norepinephrine transporter (NET) and dopamine transporter (DAT)) and multiple serotonin receptor subtypes. Off-target activity at serotonin receptors 2B (5-HT2B) and serotonin receptors 2C (5-HT2C), among others, has clinical relevance. Off-target activity at multiple serotonin receptor subtypes, particularly 5-HT2B and 5-HT2C, carries important clinical implications. Agonist activity at the 5-HT2B receptor has been implicated in structural cardiac changes, specifically valvular abnormalities and pulmonary vascular remodeling, which necessitate long-term cardiovascular monitoring in susceptible patients. In contrast, 5-HT2C receptor antagonism, observed with certain SSRIs, provides a plausible mechanistic explanation for the activation and weight loss reported by some patients during chronic treatment [16,30]. A growing literature (2021-2024) has revisited the 5-HT2B hypothesis in light of modern receptor pharmacology and population pharmacovigilance signals, urging cautious long-term monitoring rather than wholesale exclusion of SSRI classes [33-35].

Receptor-binding affinities (Ki values, nM) differentiate SSRI pharmacological fingerprints: SERT: escitalopram (0.8), sertraline (0.3), paroxetine (0.13), fluoxetine (0.8), citalopram (1.8), fluvoxamine (2.2); 5-HT2B: fluoxetine (5200) and sertraline (2200), with most SSRIs showing minimal affinity; 5-HT2C: fluoxetine (72) and sertraline (250), with antagonist activity contributing to weight effects; sigma-1 receptor (S1R): fluvoxamine (36) and escitalopram (>10000), explaining immunomodulatory profile differences.

These quantitative differences translate into clinical distinctions: fluvoxamine’s 300-fold higher σ₁R affinity relative to escitalopram provides a mechanistic basis for its unique immunomodulatory properties and its rationale in COVID-19 trials, while fluoxetine’s 5-HT2C antagonism (Kᵢ = 72 nM) mechanistically explains its increased association with weight loss compared with escitalopram (Kᵢ > 10,000 nM) [36-38].

**Figure 1:**
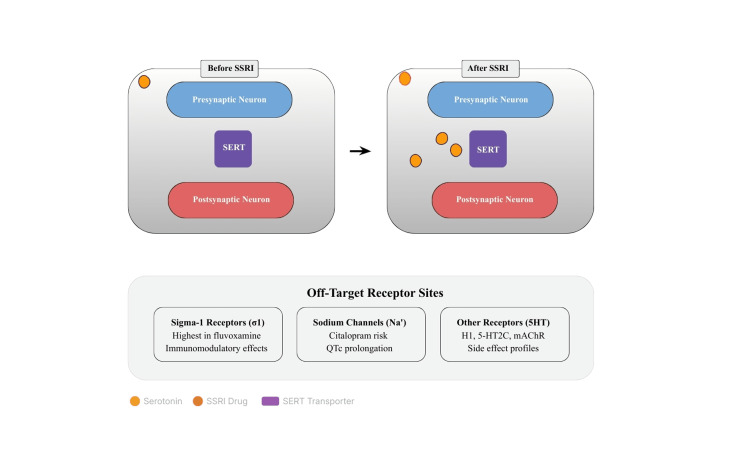
Mechanistic overview of SSRI action at the serotonin synapse The upper panel depicts neurotransmission before treatment, where serotonin is rapidly reabsorbed through SERT. The lower panel shows the post-SSRI state, where SERT inhibition increases extracellular serotonin and enhances postsynaptic activation. Off-target interactions at S1R, sodium channels (Na⁺), and serotonergic or cholinergic receptors (H1, 5-HT2C, mAChR) influence safety and tolerability, explaining inter-agent variability in adverse effects, such as QTc prolongation, sedation, and weight change. SSRI: elective serotonin reuptake inhibitor; SERT: serotonin transporter; S1R: sigma-1 receptors; H1: histamine receptor 1; 5-HT2C: serotonin receptor 2C; mAChR: muscarinic acetylcholine receptor; QTc: corrected QT interval Image credit: Author

Figure 1 also highlights several off-target receptor interactions contributing to the safety and tolerability profiles of SSRIs. These include S1R, sodium channels, and other serotonergic receptor subtypes (histamine receptor 1 (H1), serotonin receptor 2C (5-HT2C), and muscarinic acetylcholine receptor (mAChR)). S1R binding, especially in fluvoxamine, mediates immunomodulatory and potential neuroprotective effects.

Sodium channel inhibition, most relevant for citalopram, underlies its association with corrected QT interval (QTc) prolongation and conduction abnormalities [39]. Additional off-target serotonergic and histaminergic receptor interactions explain the heterogeneous side effect patterns among SSRIs, including sedation, weight changes, and agitation [40]. These off-target sites reinforce that SSRIs are not pharmacologically identical but rather form a spectrum of agents with unique receptor fingerprints and clinical consequences.

Sigma-1 Receptor, Immunomodulation, and Repurposing Signals

Some SSRIs (notably fluvoxamine and, to a lesser extent, fluoxetine/escitalopram) have a measurable affinity for the S1R, an ER chaperone that modulates cellular stress responses and cytokine production [41]. This pharmacology sparked trials of fluvoxamine in COVID-19 and mechanistic interest in anti-inflammatory and potential neuroprotective actions separate from classic SERT effects; randomized trials and meta-analyses provide preliminary evidence of reduced clinical deterioration in some outpatient COVID-19 populations, although results are heterogeneous and not sufficient to alter standard infectious disease practice beyond investigational contexts [42]. These repurposing data illustrate how off-target receptor profiles can have clinically meaningful, non-psychiatric consequences, both therapeutic and safety-related, and further support the idea that SSRIs are pharmacologically non-equivalent.

Collectively, S1R engagement may modulate neuroinflammatory cascades and oxidative stress pathways, providing a mechanistic link between psychotropic and immunomodulatory domains [36]. This receptor-mediated crosstalk remains an underexplored contributor to interindividual safety variation and therapeutic repurposing opportunities.

Ion Channel Effects and Cardiac Safety: Corrected QT Interval and Sodium Channels

SSRIs may interact with cardiac ion channels at supratherapeutic concentrations. Electrophysiology studies demonstrate dose-dependent inhibition of the cardiac sodium channel (Nav1.5) and the hERG potassium channel. Citalopram: IC₅₀ for hERG=1400-2600 nM; clinical QTc prolongation of 8-18 ms at therapeutic doses (20-40 mg). Escitalopram: IC₅₀ for hERG=2200-3800 nM; lower QTc impact (4-10 ms at 10-20 mg).

While escitalopram, the S-enantiomer of citalopram, shows significantly lower QTc prolongation potential and, in most studies, does not demonstrate clinically meaningful QTc prolongation at standard therapeutic doses. The majority of QTc risk is associated with racemic citalopram (particularly >40 mg/day). Escitalopram should still be used cautiously in high-risk populations (e.g., electrolyte imbalance, concomitant QT-prolonging drugs) [17].

Other agents can affect sodium channels and conduction at higher doses, which has informed regulatory warnings and clinical monitoring [17,40]. Contemporary population-based pharmacovigilance and ECG-monitoring studies continue to refine risk estimates and emphasize patient-level risk stratification (age, electrolyte status, concomitant QT-prolonging drugs, and congenital long-QT syndrome) [41,42].

Mechanistically, SSRI interactions with cardiac sodium (Na⁺) and hERG potassium channels alter repolarization dynamics, producing QTc prolongation and conduction delays under susceptible conditions. This electrophysiological interference, although mild at therapeutic doses, becomes clinically significant with polypharmacy or overdose, justifying ECG surveillance [17,40].

Metabolic, Endocrine, and Vascular Off-Target Consequences

Emerging evidence (2021-2024) links some SSRIs with modest perturbations in glucose and lipid metabolism, possible contributions to vascular function changes, and sexual/reproductive system effects mechanisms, likely mediated by central and peripheral receptor interactions, HPA-axis modulation, and metabolic signaling pathways [18,21]. Mechanistically, serotonergic modulation of pancreatic β-cell activity, endothelial nitric oxide pathways, and lipid metabolic enzymes provides plausible biological links between chronic SSRI exposure and subtle metabolic or vascular alterations [18,21,43]. These findings underscore the importance of monitoring metabolic parameters during long-term therapy, especially in patients with cardiometabolic risks [43].

Integrative Implication for the Mechanism Section

SSRIs act primarily via SERT inhibition but produce clinical outcomes through complex and time-dependent downstream adaptations. Off-target receptors and intracellular pharmacokinetics contribute significantly to the efficacy, tolerability, repurposing potential, and safety signals.

The integration of primary and secondary receptor targets, as summarized in Figure 1, underscores how therapeutic and adverse outcomes arise from both synaptic and extrasynaptic actions. The depiction of increased serotonin persistence after SERT blockade, alongside the influence of sigma-1 and sodium channel modulation, visually consolidates the multi-receptor complexity of modern SSRI pharmacology. The inclusion of both pre- and postsynaptic components in Figure 1 helps visualize how immediate transporter inhibition and slower downstream adaptations converge with clinical outcomes. This figure also contextualizes how receptor cross-talk, intracellular accumulation, and S1R modulation shape both therapeutic and adverse effects, supporting a multidimensional mechanistic model rather than a purely monoaminergic model [26,31]. Therefore, SSRIs should be considered pharmacologically heterogeneous agents whose selection requires attention to binding profiles, patient comorbidities, genetic/epigenetic moderators, and long-term monitoring priorities [21-27,37].

The mechanistic heterogeneity described above directly translates to clinical decision-making: the distinction between established mechanisms (SERT inhibition, receptor binding profiles confirmed by radioligand studies) and emerging hypotheses (intracellular pharmacokinetic effects, epigenetic modulation) guides both evidence-based prescribing and identification of knowledge gaps requiring further investigation. For instance, quantitative receptor binding affinities (e.g., fluvoxamine's σ₁R Ki ~36 nM vs. escitalopram's >10,000 nM) predict differential immunomodulatory and neuroprotective profiles, while SERT occupancy kinetics (>80% occupancy at therapeutic doses within hours) contrast with delayed clinical response (two to six weeks), necessitating patient education on therapeutic timelines and early tolerability management.

Translation to clinical safety is directly informed by the mechanistic heterogeneity described above. High SERT selectivity (as seen with escitalopram and paroxetine) predicts efficacy but not safety, as off-target profiles ultimately differentiate risk. Quantified receptor binding parameters (e.g., fluvoxamine's σ₁R Ki 36 nM) allow prediction of unique effects such as immunomodulation and repurposing potential. Differences between rapid SERT occupancy kinetics (>80% within hours) and delayed clinical response (two to six weeks) necessitate patient education regarding therapeutic timelines and early tolerability management. Ion channel IC₅₀ values further guide cardiac risk stratification, with citalopram doses above 40 mg requiring ECG monitoring in at-risk patients [41]. Taken together, this mechanistic framework contextualizes the clinical safety patterns, drug-drug interactions, and population-specific vulnerabilities detailed in subsequent sections.

Differentiation between selective serotonin reuptake inhibitors

Drug-Specific Variations in Receptor Affinity and Kinetics

Individual SSRI variations in pharmacokinetics and receptor binding underlie clinically meaningful differences in tolerability, safety, and interaction potential. For instance, fluoxetine’s long half-life and active metabolite, norfluoxetine, confer a low risk of discontinuation syndrome but create challenges for dose adjustments and heighten the likelihood of drug-drug interactions [21,44,45]. In contrast, paroxetine’s short half-life, high SERT potency, and strong CYP2D6 inhibition make it particularly prone to withdrawal symptoms and significant interaction potential [46-48].

Comparative Efficacy and Tolerability: Meta-Analytic Evidence

Escitalopram demonstrates the highest selectivity for SERT with minimal off-target activity. Network meta-analyses of head-to-head trials (n=21 antidepressants, 522 studies, 116477 patients) demonstrate escitalopram's superior acceptability profile, ranking highest for low discontinuation rates due to adverse effects (pooled OR vs. placebo: 0.89; 95% CI 0.77-1.03) [49]. In comparative effectiveness analyses, escitalopram exhibits a lower incidence of sexual dysfunction (30%-35%) versus paroxetine (60%-65%) and comparable efficacy (response rate OR: 1.33 vs. placebo; 95% CI 1.21-1.44) [21]. Fluvoxamine, however, shows marked S1R affinity and potent CYP1A2 inhibition, giving it unique therapeutic utility but also a distinctive interaction profile [47,48].

Sertraline’s characterization as having a "balanced profile" reflects meta-analytic evidence positioning it in the intermediate range across multiple outcome domains. Its efficacy is demonstrated by a response rate odds ratio of 1.25 (95% CI 1.15-1.37) versus placebo, performing comparably to escitalopram [49]. Tolerability data show discontinuation rates due to adverse effects of 14-18%, which fall between the lowest rates observed with escitalopram (12%) and the highest with paroxetine (22%-25%) [50]. Sexual dysfunction occurs in approximately 40%-50% of users, lower than with paroxetine but higher than with escitalopram. Sertraline also exhibits moderate CYP2D6 inhibition, less pronounced than that of paroxetine or fluoxetine, supporting its suitability in complex polypharmacy settings [46]. Overall, this "balanced" characterization reflects sertraline’s middle-ground position in the efficacy-tolerability-safety trade-offs rather than superiority in any single domain, supporting its frequent use as a first-line agent when patient-specific contraindications to escitalopram exist.

Integrative Implication for the Mechanism Section

Recent pharmacogenomic evidence suggests that CYP2C19 and CYP2D6 genotypes may partly explain inter-individual differences in SSRI plasma levels and adverse outcomes, supporting the growing role of personalized prescribing [51].

Comparative Pharmacological Profiles

Table 1 provides details of the comparative SSRI pharmacological profiles. Additionally, the SSRI mechanism section shows serotonin synapses before and after SSRI binding. Meta-analytic evidence supports these comparative tolerability claims: network meta-analyses of head-to-head trials demonstrate escitalopram's superior acceptability profile (lower discontinuation rates) compared to other SSRIs. Paroxetine consistently ranks among the agents with the highest discontinuation rates in network meta-analyses (discontinuation OR: 1.31; 95% CI 1.15-1.49 vs. placebo; highest among SSRIs alongside venlafaxine), attributable to its pronounced anticholinergic effects, high incidence of sexual dysfunction (60%-65% in naturalistic studies), and severe withdrawal symptoms upon cessation [52]. The characterization of sertraline as “balanced” reflects its intermediate position in efficacy-tolerability trade-offs across multiple outcome domains in comparative effectiveness reviews.

**Table 1:** Comparative SSRI pharmacological profiles S1R: sigma-1 receptor; SERT: serotonin transporter; SSRIs: selective serotonin reuptake inhibitors; DAT: dopamine transporter; H1: histamine receptor 1; 5-HT2C: serotonin receptor 2C; QTc: corrected QT interval

Drug	SERT inhibition potency	Other receptor affinities	Half-life	Notes
Fluoxetine	High	5-HT2C antagonist, S1R agonist	1-4 days (norfluoxetine 7-15 days)	Activating, longest duration
Paroxetine	High	Anticholinergic, minimal S1R	21 hours	Mildly sedating, high withdrawal risk
Sertraline	High	DAT blockade, S1R antagonist	26 hours	Balanced profile
Citalopram	High	Mild H1 antagonism, sodium channels	35 hours	QTc prolongation risk at doses >40 mg/day; requires ECG monitoring in at-risk patients
Escitalopram	Highest SERT selectivity	Minimal off-target effects	27-32 hours	Minimal off-target effects
Fluvoxamine	Moderate-high	Highest S1R affinity, CYP1A2 inhibition	15.6 hours	Significant drug interactions

These pharmacodynamic nuances align with the receptor and ion channel diversity illustrated in Figure 1, emphasizing that subtle differences in off-target profiles, such as S1R affinity or sodium channel inhibition, translate into measurable clinical distinctions [13,17,21,30,36,40].

Mechanistic Illustration and Off-Target Receptor Considerations

Figure 1 shows a schematic representation of serotonergic neurotransmission before and after SSRI administration. Under normal conditions, serotonin released from presynaptic neurons is rapidly cleared from the synaptic cleft through reuptake by the SERT. After SSRI treatment, blockade of SERT results in elevated extracellular serotonin levels, prolonging serotonergic signaling and modulating postsynaptic receptor activity [21-24]. However, this process extends beyond the simple inhibition of transporters. SSRIs display variable off-target binding that influences both efficacy and adverse effect profiles. Notably, S1R interaction is most pronounced with fluvoxamine and, to a lesser extent, with fluoxetine and escitalopram, conferring immunomodulatory and anti-inflammatory effects, supporting the potential repurposing of this receptor in inflammatory and viral illnesses [13,36-38]. Additionally, sodium channel interference, particularly with citalopram and escitalopram, contributes to QTc prolongation and necessitates dose-dependent cardiac monitoring [17,42]. SSRIs also interact with other serotonergic and non-serotonergic receptors (H1, 5-HT2C, mAChR), explaining the variability in sedation, weight change, and sexual dysfunction across agents [16,21,30]. These receptor- and ion-channel level differences explain the pharmacological properties of SSRIs, which are pharmacologically heterogeneous, despite sharing a common mechanism of SERT inhibition. The integration of these off-target mechanisms into safety evaluations is critical for predicting individual tolerability and optimizing drug selection [21-27,37].

Clinical safety profile

Common Adverse Effects

The most frequent adverse effects associated with SSRI therapy include sexual dysfunction, GI symptoms, cognitive slowing, and sleep disruption, which collectively affect patient quality of life and treatment adherence. A naturalistic observational study documented the high prevalence of early-onset effects, with flatulence affecting 64% of patients, somnolence reported in 59%, and cognitive symptoms, including memory impairment (51%) and reduced concentration (50%), emerging within the first weeks of treatment. Sexual adverse effects (including reduced libido, delayed ejaculation, anorgasmia, and erectile dysfunction), which affect between 30% and 70% of patients depending on the specific agent and assessment methodology, remain one of the most troubling and persistent adverse effects [52]. Notably, delayed ejaculation associated with SSRIs can provide therapeutic benefit in patients with premature ejaculation, representing a context where this adverse effect profile becomes clinically advantageous and is sometimes exploited for off-label use. Sexual adverse effects remain the leading cause of treatment discontinuation, mediated by serotonergic inhibition of dopaminergic and spinal reflex pathways [53,54].

Beyond GI side effects (nausea, diarrhea, and abdominal pain via 5-HT3 receptor stimulation, neurocognitive adverse events represent under-recognized but clinically significant tolerability concerns supported by multiple lines of evidence [55].

Prospective cohort studies with standardized batteries: Sayyah et al. [53] employed comprehensive neuropsychological testing (including the Wechsler Memory Scale and Stroop Color-Word Test) in 61 patients with depression or OCD receiving SSRI monotherapy. Following SSRI discontinuation, patients demonstrated statistically significant improvements in verbal memory (Cohen's d=0.32, p=0.03) and Stroop interference scores (p=0.02), indicating resolution of treatment-associated cognitive deficits. These findings remained significant after controlling for changes in depression severity, suggesting direct medication effects rather than disease confounding [53].

Large-scale patient-reported outcomes: A multinational cross-sectional survey (n=1,431 SSRI users) documented a high prevalence of cognitive complaints, with memory impairment reported by 51%, reduced concentration by 50%, and emotional blunting by 46% of respondents. Notably, emotional blunting emerged as the single most troubling adverse effect, rated above sexual dysfunction, and was independently associated with treatment discontinuation decisions [56].

Meta-analytic evidence: Recent meta-regression analyses of randomized controlled trials, incorporating data from prospective cohort studies using propensity score matching to control for confounding by indication, have identified small but statistically significant dose-dependent deficits in verbal memory (Cohen's d=0.2-0.3, 95% CI: 0.15-0.35) and processing speed during acute SSRI treatment. These effects persist after adjustment for baseline depression severity and demonstrate temporal correlation with plasma SSRI concentrations [53,57].

Mechanistic correlates: Cognitive adverse effects correlate with serotonergic interference in cholinergic pathways, particularly through serotonin receptor 2A (5-HT2A) receptor overstimulation in prefrontal cortex regions critical for working memory and executive function [56]. Comparative studies demonstrate that SSRIs with higher anticholinergic burden (paroxetine) produce greater cognitive impact than highly selective agents (escitalopram), supporting receptor-specific mechanisms [30,55].

These convergent findings across multiple study designs, including prospective cohorts with objective cognitive assessment, large-scale naturalistic surveys, and meta-analytic syntheses, establish neurocognitive adverse effects as a reproducible SSRI class effect requiring clinical monitoring, particularly in elderly patients, those with baseline cognitive vulnerability, and individuals in cognitively demanding occupations.

Sleep and Neuropsychiatric Effects

SSRIs exert heterogeneous sleep effects, with fluoxetine and sertraline generally activating sleep, while paroxetine remains sedating and is sometimes leveraged in patients with comorbid insomnia [21,57]. This paradox reflects the dual role of serotonin in initiating sleep and serving as a precursor of melatonin.

Neuropsychiatric effects, such as headaches, agitation, and emotional blunting, have been increasingly reported. Overstimulation of 5-HT2A receptors has been linked to headaches, whereas serotonergic-cholinergic interplay may explain memory impairments [58]. Notably, recent real-world pharmacovigilance studies have confirmed higher reporting of emotional blunting and apathy with paroxetine and fluoxetine than with escitalopram [59].

Other Adverse Events of Interest

QTc prolongation and associated cardiac conduction abnormalities remain most pronounced with citalopram, a concern that has led regulatory agencies, including the FDA, to impose strict dose restrictions, particularly in older adults and those with pre-existing cardiac vulnerability [22]. Emerging pharmacovigilance data indicate that polypharmacy substantially amplifies these risks, with concurrent use of antipsychotics or macrolide antibiotics creating additive QTc effects that increase the likelihood of potentially fatal ventricular arrhythmias [60]. SIADH and consequent hyponatremia represent particularly problematic complications in elderly patients, where age-related physiological changes and concurrent diuretic therapy create a perfect storm for electrolyte disturbances. Among SSRIs, citalopram and escitalopram carry the highest risk for inducing clinically significant hyponatremia [61]. Suicidality continues to be a concern in individuals under 25 years of age, with meta-analyses confirming elevated early treatment risk, likely due to activation before the therapeutic benefit emerges [62]. Serotonin syndrome is rare but severe. Recent pharmacovigilance analyses have revealed that fluvoxamine and paroxetine carry the highest reporting odds ratios (ROR) [63].

Withdrawal and Discontinuation Syndrome

Withdrawal remains a significant concern, with symptoms such as dizziness, “electric shocks,” and anxiety. Over 31000 SSRI withdrawal cases have been reported globally, predominantly in women, with symptom onset 36-96 h post-cessation [64]. Paroxetine carries the highest risk because of its short half-life, whereas fluoxetine carries the lowest risk [65].

Recent systematic reviews have confirmed that withdrawal may persist for months or years, with online patient-reported outcomes indicating longer durations for SSRIs than for SNRIs [66,67]. These findings reinforce the need for structured tapering protocols and patient education.

Population-Specific Risks

Elderly patients face elevated risks of falls, hyponatremia, and QTc prolongation due to pharmacokinetic changes and polypharmacy [53,68].

Pregnancy and neonates: First-trimester exposure to paroxetine and fluoxetine is associated with small but significant increases in cardiac malformations, whereas third-trimester exposure increases the risk of PPHN and neonatal adaptation syndrome [69-71].

Youth and adolescents: Suicidal ideation risk remains highest under the age of 25, necessitating close monitoring and integration of psychosocial interventions. Table 2 provides details of population-specific adverse event profiles [72].

**Table 2:** Population-specific adverse event profiles SSRIs: selective serotonin reuptake inhibitors; SIADH: syndrome of inappropriate antidiuretic hormone secretion; QTc: corrected QT interval

Adverse effect	Frequency (approx.)	SSRI(s) most often implicated	Key risk factors (population/drug)	References
Sexual adverse effects (libido, orgasm, and arousal domains)	Approximately30%-70%, depending on agent, assessment method, and population (paroxetine often highest)	Paroxetine>sertraline≈fluoxetine (paroxetine reported ~60%-65%)	Higher dose, longer duration, male sex for some outcomes; depression itself also causes sexual dysfunction	[55]
QTc prolongation/torsades risk (drug-induced)	Low-moderate overall; dose-dependent with citalopram, clinically relevant risk at higher doses	Citalopram, escitalopram (citalopram's strongest signal)	Dose >40 mg/day (citalopram), advanced age, electrolyte disturbances (hypokalemia/magnesaemia), other QT-prolonging drugs, and congenital long QT	[68]
Hyponatremia (SIADH-like)	Range: short-term ~0.9% (30 days) to ~10% over longer follow-up in some cohorts; elderly show higher rates (several %)	Citalopram, escitalopram, other SSRIs (signals across class; some studies show higher rates with particular agents)	Older age (≥65), concomitant thiazide diuretics, low baseline sodium, low body weight, and comorbidities	[69,70]
Withdrawal/discontinuation syndrome	Approximately 15%-56%, depending on definition and drug; paroxetine shows the highest rates	Paroxetine, venlafaxine, desvenlafaxine (highest); fluoxetine lower risk due to long half-life	Short half-life agents, abrupt cessation or rapid taper, higher dose, and longer exposure	[70,71]
Treatment-emergent suicidal ideation/behavior (children/young adults)	Variable-trials showed ~4% events on drug vs ~2% on placebo in youth; absolute risk small but relative increase observed in <25 yrs	All SSRIs (class warning applies)	Age <25 (highest signal), early treatment period (first weeks to months), prior suicide history	[72]

Cardiac Safety Considerations

Among SSRIs, citalopram and to a lesser extent escitalopram demonstrate dose-dependent QTc interval prolongation, mediated by blockade of the hERG (human ether-à-go-go-related gene) potassium channel. Following postmarketing surveillance identifying cases of torsades de pointes, the FDA issued safety communications in 2011-2012 restricting maximum citalopram doses to 40 mg/day in adults and 20 mg/day in patients over 60 years of age, those with hepatic impairment, or CYP2C19 poor metabolizers [41,42]. ECG monitoring is recommended in patients with predisposing risk factors, including structural heart disease, electrolyte disturbances (hypokalemia and hypomagnesemia), concomitant QT-prolonging medications, or congenital long QT syndrome. Despite these restrictions, the absolute risk of clinically significant arrhythmias remains low in the absence of multiple risk factors, and citalopram/escitalopram continue to demonstrate favorable cardiovascular safety profiles compared to TCAs. Other SSRIs (fluoxetine, sertraline, paroxetine, and fluvoxamine) show negligible effects on cardiac conduction parameters at therapeutic doses [60,68]. Population-specific risk stratification is critical for safe SSRI prescribing. Sexual adverse effects affect 30%-70% of patients depending on agent and assessment method, with paroxetine demonstrating the highest incidence (~60%-65%) [55]. QTc prolongation risk with citalopram is dose-dependent and amplified in elderly patients (≥65 years), those with electrolyte abnormalities, and concomitant QT-prolonging medications [66]. Hyponatremia, presenting as SIADH-like syndrome, occurs in approximately 0.9% of patients within 30 days of SSRI initiation, but rates approach 10% in longer-term elderly cohorts, particularly when combined with thiazide diuretics [69,70]. Withdrawal syndromes affect 15%-56% of patients depending on definition and agent, with paroxetine and venlafaxine showing the highest rates due to short half-lives, while fluoxetine demonstrates protective effects (due to its long-acting metabolite norfluoxetine) [70,71]. Treatment-emergent suicidal ideation in youth (<25 years) shows approximately 4% event rate with SSRIs versus 2% with placebo, with the highest risk in the early treatment period [72].

Pharmacovigilance and regulatory insights

Analysis of global pharmacovigilance databases including WHO VigiBase (>35 million individual case safety reports from >150 countries) and FAERS (>18 million reports through 2024) confirms specific SSRI-adverse event associations through rigorous disproportionality analyses [19,73].Quantitative signal detection using RORs with 95% confidence intervals reveals the following safety signals: serotonin syndrome, with pooled RORs across SSRIs ranging from 15.2 to 24.8 (95% CIs vary by agent), the highest signals observed for fluoxetine (ROR 24.8; 95% CI 21.3-28.9) and fluvoxamine (ROR 23.1; 95% CI 18.4-29.0), and substantial risk amplification with concomitant serotonergic agents, particularly SSRI plus linezolid (ROR 392.9; 95% CI 312.5-493.8), reflecting dangerous pharmacodynamic interactions; hyponatremia, with class-wide RORs ranging from 3.2 to 7.8 depending on database and population, the highest signals observed for escitalopram (ROR 7.8; 95% CI 6.9-8.8) and citalopram (ROR 6.4; 95% CI 5.8-7.1), and age-stratified risk demonstrating that patients ≥65 years have an ROR of 12.3 (95% CI 10.1-15.0), confirming the elderly as a high-risk population; hemorrhagic events, including upper GI bleeding with RORs of 1.5-2.5 for SSRI monotherapy escalating to 4.2-6.8 with concomitant NSAID or anticoagulant use, and intracranial hemorrhage with drug-specific patterns identified, sertraline (ROR 2.3; 95% CI 2.0-2.7) and citalopram (ROR 2.8; 95% CI 2.3-3.4), reflecting serotonergic effects on platelet function; sexual dysfunction, with RORs ranging from 8.2 to 15.6 across SSRIs, although marked underreporting is evident when compared with prospective studies reporting 30%-70% prevalence, including PSSD with an ROR of 11.4 (95% CI 8.9-14.6), predominantly reported for paroxetine and sertraline; discontinuation/withdrawal syndrome, with agent-specific RORs showing the highest risk for paroxetine (ROR 7.3; 95% CI 6.8-7.9) and venlafaxine (ROR 8.1; 95% CI 7.5-8.8), contrasted with a protective effect observed for fluoxetine (ROR 0.32; 95% CI 0.28-0.37), consistent with its long elimination half-life and active metabolite norfluoxetine; and akathisia, with RORs ranging from 4.8 to 12.6 across individual SSRIs, the highest signals observed for fluoxetine (ROR 12.6; 95% CI 9.8-16.2) and sertraline (ROR 9.4; 95% CI 7.6-11.6) [10,18,61,74-82].

These quantitative disproportionality analyses, derived from millions of real-world adverse event reports, confirm that while SSRIs share a common mechanism of serotonin reuptake inhibition, individual agents demonstrate statistically distinct safety profiles. The magnitude and precision of these RORs (reflected in narrow confidence intervals for high-frequency events) provide robust signals that inform personalized prescribing decisions based on patient-specific risk factors, including age, concomitant medications, and baseline comorbidities.

Regulatory activities, including Food and Drug Administration and European Medicines Agency actions

**Figure 2:**
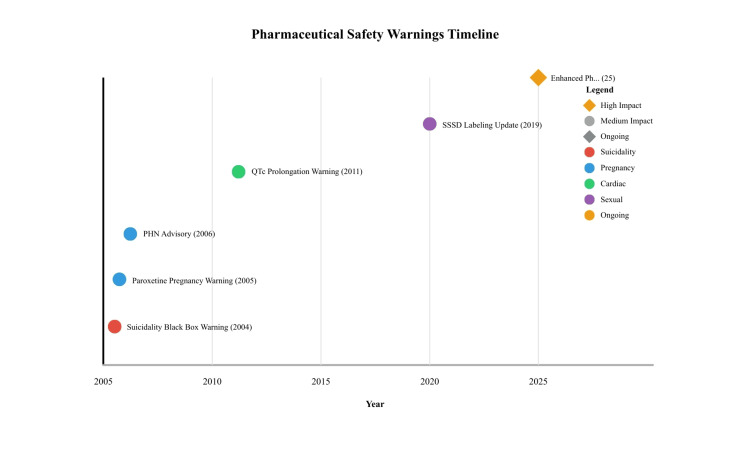
Timeline of major regulatory actions related to SSRIs SSRIs: selective serotonin reuptake inhibitors; QTc: corrected QT interval: PHN: postherpetic neuralgia; SSSD: SSRI-associated sexual dysfunction Image credits: Author

Regulatory actions have evolved in response to emerging safety concerns. In 2004, the FDA introduced a black box warning for suicidality in children based on a meta-analysis of pediatric SSRI trials. This action led to decreased prescriptions in younger populations and increased monitoring of adverse effects [79]. In 2011, the FDA imposed dose limits on citalopram due to the risk of QTc prolongation: 40 mg/day for adults and 20 mg/day for those aged ≥ 60 years, along with ECG monitoring for high-risk patients [21]. In 2019, the European Medicines Agency (EMA) mandated labeling changes regarding PSSD, where the Pharmacovigilance Risk Assessment Committee (PRAC) required warnings about the potential for lasting sexual side effects to be included in package leaflets [83]. In addition, post-2020 pharmacovigilance analyses have emphasized the underreporting of long-term neuropsychiatric adverse effects, underscoring the gaps in pre-marketing trial designs and post-marketing safety strategies (Figure 2) [84,85]. These regulatory measures illustrate a largely reactive approach, highlighting the ongoing need for proactive signal detection and earlier translation into clinical guidance [50]. Table 3 provides major regulatory actions.

**Table 3:** Major regulatory actions PPHN: persistent pulmonary hypertension of the newborn; PSSD: post-SSRI sexual dysfunction; SSRIs: selective serotonin reuptake inhibitors; FDA: Food and Drug Administration; FAERS: FDA Adverse Event Reporting System; EMA: European Medicines Agency; QTc: corrected QT interval

Event	Year	Drug(s) affected	Regulatory action	Data source
Suicidality warning	2004	All SSRIs	Black Box for under 24	FDA meta-analysis
Paroxetine pregnancy warning	2005	Paroxetine	Category D classification	Birth defects registries
PPHN advisory	2006	All SSRIs	Late pregnancy warning	Case reports, epidemiology
QTc warning	2011	Citalopram	Max dose limit 40 mg	FAERS, clinical studies
PSSD labeling	2019	All SSRIs	Product labeling update	EMA pharmacovigilance

Drug-drug interactions and metabolic concerns

Pharmacokinetic Interactions

Anticoagulant and antiplatelet effects: The risk of bleeding increases by approximately 40%-70% among patients taking SSRIs, with the greatest elevations observed when these agents are combined with anticoagulants such as warfarin, antiplatelet agents like aspirin, or nonsteroidal anti-inflammatory drugs (NSAIDs). This synergistic effect arises through dual mechanisms: alterations in drug metabolism and direct inhibition of platelet serotonergic function [35]. For example, fluoxetine significantly slows warfarin metabolism through potent inhibition of the cytochrome P450 enzyme CYP2C9, leading to elevated anticoagulant concentrations that enhance anticoagulation beyond therapeutic targets and substantially increase bleeding risk. Recent meta-analyses confirm higher upper GI bleeding risk with SSRIs co-prescribed with NSAIDs or DOACs, especially in elderly patients [77,86]. Sertraline, citalopram, and escitalopram have less interaction potential and are generally safer in anticoagulated patients.

Tamoxifen interactions: Tamoxifen requires CYP2D6 metabolism to form active endoxifen. Paroxetine and fluoxetine strongly inhibit this pathway, potentially reducing anticancer efficacy [77]. With patients on tamoxifen, citalopram, escitalopram, and sertraline are favored. Updated oncology guidelines continue to caution against strong CYP2D6 inhibitors in breast cancer survivors, citing robust evidence of treatment compromise [87].

Opioid metabolism effects: SSRIs also alter opioid metabolism via CYP450 pathways. Both paroxetine and fluoxetine inhibit the activation of codeine to morphine by CYP2D6, leading to reduced analgesia and accumulation of parent compounds, raising the risk of respiratory depression [77]. Recent pharmacoepidemiologic data also suggest that SSRI-opioid co-use may increase the risk of opioid-related hospitalization in older adults [88]. Major SSRI drug interactions are given in Table 4.

**Table 4:** Major SSRI drug interactions SSRIs: selective serotonin reuptake inhibitors; QTc: corrected QT interval

SSRI	Major CYP inhibited	Affected medications	Interaction outcome	Interaction severity
Fluoxetine	CYP2D6, CYP2C9	Tamoxifen, warfarin, β-blockers	Reduced efficacy, ↑ bleeding	Major
Paroxetine	CYP2D6 (potent)	Tamoxifen, codeine, metoprolol	Reduced efficacy, altered metabolism	Major
Fluvoxamine	CYP1A2, CYP2C19	Theophylline, caffeine, diazepam	Toxicity risk, ↑ concentrations	Major
Sertraline	CYP2D6 (weak)	Minimal significant interactions	Generally safer profile	Minor-moderate
Citalopram	Minimal	QTc-prolonging drugs	Additive cardiac effects	Moderate-major
Escitalopram	Minimal	QTc-prolonging drugs	Additive cardiac effects	Moderate

Elderly and Complex Patients: Clinical Implications for Polypharmacy

The management of SSRI therapy in elderly patients and those with complex medication regimens presents substantial challenges that are increasingly well-documented through comprehensive pharmacovigilance analyses. According to robust pharmacovigilance data examining drug-drug interaction signals, certain SSRI combinations carry exceptionally high risks that demand heightened clinical vigilance and, when possible, therapeutic alternatives. Most notably, the concurrent use of SSRIs with linezolid, a MAOI antibiotic, generates an extraordinarily elevated ROR of 392.90, reflecting the profound serotonergic potentiation and risk of life-threatening serotonin syndrome [80]. Similarly, combinations with traditional MAOIs produce a ROR of 124.15, while SSRI-opioid combinations yield a ROR of 41.95, both representing clinically significant interaction risks [80]. These quantified risk estimates underscore the imperative to avoid such combinations whenever therapeutically feasible alternatives exist, and when concurrent use cannot be avoided, to implement intensive monitoring protocols.

Beyond these acute high-risk combinations, the broader context of polypharmacy in psychiatric care introduces additional layers of complexity, particularly concerning treatment discontinuation. Pharmacoepidemiologic evidence reveals that concurrent psychotropic medication use dramatically amplifies the risk of SSRI withdrawal syndromes, with patients taking multiple psychotropic agents facing more than six times the odds of experiencing withdrawal symptoms (OR 6.21) compared to those on SSRI monotherapy [81]. This elevated risk manifests differentially across drug classes, with antipsychotic co-prescription conferring a more than three-fold increase (OR 3.28), benzodiazepine co-use nearly doubling the risk (OR 1.99), and mood stabilizer combinations increasing odds by 93% (OR 1.93) [81].

Gaps in the literature and research needs

Under-Explored Adverse Effects

Long-term metabolic effects: Accumulating evidence suggests potential correlations between chronic SSRI exposure and metabolic disturbances, including weight gain, insulin resistance, and dyslipidemia, though establishing definitive causality remains challenging given the confounding effects of depression itself on metabolic parameters. A comprehensive prospective cohort meta-analysis published in 2023 (n=12 studies, >45000 participants, mean follow-up 3.2 years) employed multiple confounding control strategies including: (1) propensity score matching on baseline depression severity, BMI, and comorbidities; (2) time-varying covariate adjustment for depression trajectory during follow-up; and (3) sensitivity analyses comparing SSRI users to both untreated depressed controls and patients treated with non-metabolically active antidepressants (e.g., bupropion). After these rigorous adjustments, users of paroxetine and escitalopram exhibited significantly higher odds of developing metabolic syndrome compared to matched controls (adjusted OR 1.37; 95% CI 1.18-1.59 for paroxetine; adjusted OR 1.24; 95% CI 1.09-1.42 for escitalopram), with dose-response relationships evident in stratified analyses. Notably, the association persisted in analyses restricted to patients with remitted depression, suggesting medication effects independent of active depressive symptoms [65,87].

These findings highlight an urgent need for systematic long-term cardiometabolic surveillance in patients maintained on SSRI therapy, particularly those with pre-existing metabolic risk factors. Recommended monitoring includes: baseline and annual assessment of weight, waist circumference, fasting glucose, lipid panel, and blood pressure in patients on long-term SSRI therapy (>1 year), with consideration of switching to metabolically neutral alternatives (e.g., fluoxetine, sertraline at lower doses) in patients developing metabolic syndrome components.

Neurocognitive consequences: Cognitive effects of chronic SSRI use remain critically underexplored despite patient-reported concerns. Large-scale surveys consistently document high prevalence of memory impairment (51%) and concentration difficulties (50%) among SSRI users [54,82]. Moving beyond cross-sectional associations, recent longitudinal cohort analyses have attempted to establish temporal relationships between prolonged SSRI exposure and dementia risk in older adults using sophisticated epidemiological designs. The methodological approaches employed included propensity score matching, in which SSRI-exposed patients (n ≈ 80,000) were matched to unexposed depressed controls on more than 50 baseline covariates, including age, sex, depression severity, comorbidities, socioeconomic status, and baseline cognitive function, typically assessed using claims-based algorithms or registry cognitive scores; time-varying confounding adjustment using Cox proportional hazards models incorporating time-updated depression status, SSRI exposure windows, and competing risks such as death; multiple imputation to handle missing covariate data, present in approximately 15-30% of registry records, using chained equations under missing-at-random assumptions; and sensitivity analyses comprising restriction to new-user designs to avoid prevalent-user bias, implementation of lag periods of 1-2 years to address reverse causation (prodromal dementia → depression → SSRI), and use of negative control outcomes, such as fractures, to detect unmeasured confounding.

Despite these rigorous methods, findings remain inconsistent. Some studies report modest associations between prolonged SSRI exposure (>5 years cumulative use) and increased dementia risk (adjusted HR 1.12-1.29), while others find null results or even protective effects. Key methodological challenges limiting causal inference include confounding by indication, whereby depression itself is a robust risk factor for dementia (hazard ratio approximately 2.0) and residual confounding persists despite the use of propensity score methods; immortal time bias, in which patients must survive depression long enough to accumulate extended SSRI exposure, thereby introducing survivor bias; detection bias, as SSRI users have more frequent healthcare encounters, increasing the likelihood of dementia diagnosis; and time-varying confounding, given that depression severity fluctuates over time, complicating exposure-outcome relationships.

To definitively establish causality, future research requires: (1) randomized trials of SSRI continuation versus discontinuation in older adults with remitted depression, with cognitive outcomes as co-primary endpoints; (2) biomarker-enriched cohorts combining longitudinal neuroimaging (volumetric MRI), fluid biomarkers (Aβ42, tau, neurofilament light), and cognitive testing; and (3) pharmacoepidemiologic studies leveraging instrumental variable approaches (e.g., prescriber preference, genetic variants affecting SSRI metabolism) to address unmeasured confounding [66,89].

Sexual adverse effects: SSRI-induced sexual dysfunction can persist as PSSD. In 2024, a multicenter prospective cohort study with active surveillance and validated sexual function instruments reported persistent genital numbness and anorgasmia in 13.2% of young adult SSRI users, emphasizing the need for standardized diagnostic tools and mechanistic studies [66].

Akathisia and movement disorders: Akathisia, often underdiagnosed, occurs in up to 25% of SSRI users [86]. Cross-sectional pharmacovigilance studies employing disproportionality analysis highlight its strong association with treatment discontinuation and suicidal behaviors, calling for wider use of the Barnes Akathisia Rating Scale in both clinical trials and practice [90,91].

Cardiovascular pathologies: Beyond neuromotor adverse events, SSRIs may contribute to cardiovascular pathologies via 5-HT2B receptor agonism, linked to valvular disease and pulmonary hypertension [92]. Emerging preclinical evidence underscores the relevance of receptor-specific off-target effects, suggesting opportunities for the development of safer serotonergic agents to reduce SSRI-induced akathisia [93].

Population-Specific Research Needs

Geriatric considerations: The prescription of SSRIs in older adults presents unique clinical challenges that extend beyond simple dose adjustment, encompassing age-related pharmacokinetic alterations, extensive polypharmacy, and multiple medical comorbidities that collectively increase vulnerability to adverse effects such as hyponatremia, falls, and clinically significant drug-drug interactions. Despite the widespread use of SSRIs in geriatric populations and the well-documented risks they face, there remains a concerning paucity of adequately powered prospective clinical trials specifically designed to define optimal dosing regimens, establish therapeutic windows, and develop evidence-based monitoring protocols tailored to the unique physiological characteristics and clinical contexts of elderly patients [55,73]. Recent observational studies suggest that SSRI initiation in older adults is associated with small but significant increases in risk of falls, osteoporosis, and hyponatremia, warranting dose adjustment guidelines and post-marketing surveillance in elderly cohorts [83,94].

Because neurodegenerative changes (e.g., in Alzheimer’s disease, Parkinson’s) alter serotonergic signaling and receptor density with age, SSRIs’ interactions with these altered circuits merit a mechanistic study [95]. Longitudinal studies combining neuroimaging and biomarker assays are needed to clarify whether SSRIs affect the progression of neurodegeneration or cognitive decline in older patients [96].

Pregnant women and offspring: While short-term safety data in pregnancy exist, long-term neurodevelopmental outcomes in exposed offspring remain underexplored. Some large registry studies report modest associations between prenatal SSRI exposure and neurodevelopmental disorders (autism spectrum disorder, ADHD), but confounding by maternal illness is pervasive [69,97]. Future research should stratify by maternal genetics, fetal sex, exposure timing, SSRI dose, and adjust for maternal depression severity and environmental factors. Prenatal precision-medicine approaches requiring maternal pharmacogenomic profiling, placental transporter genotyping, and fetal biomarkers could illuminate vulnerability windows and guide safer prescribing during gestation [98].

Adolescents and young adults: Although suicidality risk is acknowledged in patients < 25, most safety trials systematically exclude high-risk youth (e.g., with suicidality or psychiatric comorbidity), limiting external validity. To fill this gap, pragmatic trials and cohort studies in real-world adolescent populations (including those with comorbidity, substance use, or prior adverse drug reactions (ADRs)) are urgently needed [99]. Further, developmental brain sensitivity in adolescence raises the possibility of long-term adverse cognitive, emotional, or reward-system sequelae following SSRI exposure. Longitudinal cohort studies and neuroimaging follow-ups are needed to elucidate these risks.

Need for Large-Scale Pharmacovigilance Database Studies and Integrated Data Platforms

Current pharmacovigilance systems rarely integrate genomic or biomarker data, limiting their ability to detect genetically vulnerable subgroups. Linking pharmacogenomic data (e.g., CYP2D6, SLC6A4 polymorphisms, and methylation status) with adverse event databases could enable predictive safety modeling and risk stratification [93,100]. The application of machine learning to electronic health records, registries, insurance claims, and patient-generated digital data presents an opportunity to enhance the detection of rare or complex safety signals that randomized trials cannot capture [101]. Patient-reported outcomes (via apps, wearables) may uncover withdrawal, emotional blunting, or quality-of-life effects underrecognized in clinical trials.

WHO VigiBase and global pharmacovigilance: The WHO VigiBase, recognized as the world's largest and most comprehensive database of individual case safety reports, serves as an invaluable global repository for documenting and analyzing adverse events associated with SSRI therapy [102]. This extensive pharmacovigilance platform, which aggregates spontaneous adverse event reports from over 150 countries, provides unique opportunities for advancing our understanding of SSRI safety beyond the limitations inherent in traditional randomized controlled trials. Leveraging the vast scope and granular detail contained within VigiBase enables researchers and regulatory agencies to detect rare or delayed adverse events that typically remain unidentified during pre-marketing clinical trials due to limited sample sizes and relatively short follow-up periods. Furthermore, the database's comprehensive coverage across multiple SSRIs facilitates robust comparative safety profiling, allowing clinicians to make evidence-based decisions when selecting among different agents within this therapeutic class based on their distinct risk profiles. The inclusion of detailed demographic and clinical data within VigiBase also permits sophisticated stratified analyses that can identify vulnerable subpopulations, examining how adverse event patterns vary by critical factors such as age, sex, underlying comorbidities, and concomitant medication use [49].

The true translational potential of VigiBase emerges when this pharmacovigilance data is systematically integrated with complementary data sources, including population-level prescribing patterns, pharmacogenomic information, and detailed clinical metadata from electronic health records. Such multi-dimensional data integration creates a powerful framework for transforming raw safety signals into actionable, evidence-based prescribing guidelines that can be implemented at the point of care. By combining the broad surveillance capabilities of VigiBase with targeted genomic profiling and real-world clinical data, the field can move toward a precision pharmacovigilance approach that not only identifies risks at the population level but also predicts individual patient vulnerability, ultimately enabling more personalized and safer therapeutic strategies in psychiatric care.

Recommendations for future studies

Standardized Adverse Drug Reaction Definitions

A fundamental impediment to advancing SSRI safety research lies in the inconsistent terminology and definitions applied to adverse events across different studies, with particularly problematic ambiguity surrounding concepts such as withdrawal versus discontinuation syndrome. Achieving standardization in adverse event reporting represents a critical priority that requires coordinated international effort. The widespread adoption of validated, quantitative rating scales, such as the Barnes Akathisia Rating Scale for movement disorders and the Arizona Sexual Experiences Scale for sexual dysfunction, coupled with harmonized diagnostic criteria based on MedDRA terminology, would dramatically improve the comparability of findings across studies and enable more robust meta-analytic synthesis of safety data [49,79]. These efforts should be operationalized through longitudinal cohort designs that track adverse events prospectively over extended periods, complemented by Bayesian pharmacovigilance models that integrate multiple data sources (spontaneous reporting systems, electronic health records, and clinical trial registries) to detect signals earlier and estimate event probabilities with greater precision.

Methodological Frameworks for Operationalization

To translate these recommendations into actionable research, specific methodological approaches should be prioritized.

Longitudinal cohort designs include population-based inception cohorts with a minimum 10-year follow-up that recruit patients at SSRI initiation to minimize immortal time bias and survivor effects; repeated-measures designs with quarterly assessments during years 1-2 and biannual assessments thereafter to capture the temporal dynamics of adverse events; active surveillance protocols using validated instruments (Barnes Akathisia Scale, ASEX, and PHQ-9) administered systematically rather than relying on spontaneous reporting; and nested case-control substudies within cohorts to evaluate genetic and epigenetic moderators (CYP2D6, SLC6A4, methylation patterns) [22,98].

Bayesian pharmacovigilance models encompass multi-parameter Bayesian networks that integrate spontaneous reports (VigiBase, FAERS), electronic health records, prescription databases, and genetic registries; the use of Bayesian shrinkage priors for rare-event signal detection to address sparse data and false discovery rates inherent in traditional disproportionality analyses; temporal pattern mining via Bayesian change-point detection to differentiate early- versus late-onset adverse events and identify critical exposure windows; and multi-level models that account for hierarchical data structures, such as patients nested within prescribers or regions, while adjusting for confounding by indication [100,102].

Pragmatic randomized controlled trials include registry-based randomized trials embedded within routine clinical care, which minimize selection bias while maintaining causal inference, as well as Sequential Multiple Assignment Randomized Trials (SMART) designs that evaluate adaptive treatment strategies and discontinuation protocols [93].

Machine learning applications include the use of random forest and gradient boosting algorithms for adverse event prediction incorporating more than 100 baseline features, such as demographics, comorbidities, concomitant medications, and pharmacogenomic data; natural language processing of clinical notes to detect under-documented adverse events, including emotional blunting and cognitive changes; and causal inference frameworks, such as doubly robust estimation and targeted maximum likelihood, to adjust for time-varying confounding in observational data [101].

These frameworks address core methodological challenges confounded by indication, measurement error, selection bias, and sparse data while leveraging emerging data sources (wearables, patient-reported outcomes via apps) to capture real-world safety signals with greater precision than traditional trial designs.

Long-Term Safety Surveillance

Large-scale cohort studies with five to 10 years of follow-up are needed to assess chronic outcomes, including metabolic syndrome, cardiovascular pathology, persistent sexual dysfunction, and cognitive decline. The natural history of SSRI tapering, predictors of relapse or successful cessation, and individual trajectory modeling remain significant gaps.

Inclusion of Real-World Populations

Trials must reflect real-world complexity, including patients with multiple comorbidities, polypharmacy, substance use disorders, and diverse demographic backgrounds (ethnic minorities, older adults, pregnant women, and adolescents) [55]. Coupling these trials with real-world safety datasets (e.g., VigiBase, national claims, and EHRs) enables detection of population-specific safety signals and temporal patterns (early vs. late-onset AEs).

Mechanistic and Biomarker Research

Mechanistic and biomarker-driven investigations represent a critical frontier in advancing our understanding of SSRI safety and optimizing individualized treatment approaches. Research priorities should emphasize the identification and validation of genetic and epigenetic predictors that can forecast which patients are most vulnerable to withdrawal symptoms, serious adverse events, or poor tolerability. Specific molecular targets warranting investigation include transporter gene polymorphisms, particularly in the SERT gene (SLC6A4), and epigenetic modifications such as promoter methylation signatures that may modulate drug response and adverse event susceptibility [22].

Equally important is the development and clinical implementation of biomarkers capable of detecting or predicting serious adverse events before they manifest clinically or progress to irreversible harm. Promising candidates include inflammatory markers that may signal emerging metabolic or cardiovascular complications, platelet serotonin metabolites that could identify patients at heightened bleeding risk, and electrocardiographic or ion channel biomarkers that might predict cardiac conduction abnormalities and arrhythmic potential [77].

Advanced neuroimaging approaches offer unprecedented opportunities to visualize and quantify brain changes associated with chronic SSRI exposure and withdrawal. Longitudinal neuroimaging studies incorporating structural MRI, functional connectivity analyses, and potentially molecular imaging techniques should be prioritized to track adaptive and potentially maladaptive neural circuit changes during long-term SSRI use and throughout the withdrawal process, with particular emphasis on vulnerable populations such as adolescents, elderly patients, and those with pre-existing neuropsychiatric comorbidities.

Finally, the integration of complex patient-level data through sophisticated pharmacokinetic and pharmacodynamic modeling represents a path toward precision psychiatry. These models should incorporate multiple determinants of drug exposure and response, including age-related physiological changes, medical comorbidities that alter drug disposition, concurrent medications that create pharmacokinetic or pharmacodynamic interactions, and individual genetic profiles that influence drug metabolism and target receptor function. Such integrative approaches hold promise for generating personalized dosing algorithms that optimize therapeutic efficacy while minimizing individual risk profiles.

## Conclusions

SSRIs remain foundational in psychiatric pharmacotherapy, offering superior tolerability compared to older antidepressants. However, their safety profile extends beyond serotonin reuptake inhibition, shaped by off-target effects, genetic variability, and real-world prescribing complexity. Pharmacovigilance data reveal risks underrepresented in trials: bleeding (relative risk 1.4-1.7), QTc prolongation (citalopram >20 mg dose-dependent), persistent sexual dysfunction (reported prevalence 0.5-5%), akathisia, and drug-drug interactions. Regulatory responses, black box warnings for suicidality, citalopram dose restrictions, and EMA labeling for PSSD demonstrate progress but remain reactive. Advancing SSRI safety requires proactive integration of real-world evidence, pharmacogenomics, mechanistic biomarkers, and individualized prescribing strategies that account for age, comorbidities, polypharmacy, and genetic profiles. Systematic monitoring of cardiac, metabolic, cognitive, and sexual health is essential. Ultimately, balancing therapeutic benefit with long-term safety demands coordinated clinical trials, genomics research, and regulatory innovation guiding psychiatry toward precision medicine, where SSRIs remain first-line agents used with informed vigilance.

## Appendices

Appendix A

**Table 5:** MedDRA PTs and SMQs relevant to SSRI safety monitoring SMQ: standardized MedDRA query; PT: preferred term; PSSD: post-SSRI sexual dysfunction; GI: gastrointestinal; ALT: alanine aminotransferase; AST: aspartate aminotransferase; SSRIs: selective serotonin reuptake inhibitors; SIADH: syndrome of inappropriate antidiuretic hormone; PPHN: persistent pulmonary hypertension of the newborn; QTc: corrected QT interval

S.no	Safety topic	SMQ	MedDRA PTs
1	Serotonin syndrome	Serotonin syndrome (narrow)	Serotonin syndrome; neuroleptic malignant syndrome; hyperthermia malignant; myoclonus; hyperreflexia; diaphoresis; tremor; mental status changes; agitation; confusion
2	Sexual dysfunction/PSSD	Sexual function disorders (broad)	Sexual dysfunction; erectile dysfunction; ejaculation disorder; ejaculation delayed; anorgasmia; orgasm abnormal; libido decreased; genital anesthesia; sexual arousal disorder; premature ejaculation; retrograde ejaculation; vaginal lubrication decreased; persistent genital arousal disorder
3	GI bleeding	GI hemorrhage (narrow and broad)	GI hemorrhage; upper GI hemorrhage; lower GI hemorrhage; hematemesis; melaena; hematochezia; gastric hemorrhage; duodenal ulcer hemorrhage; rectal hemorrhage; peptic ulcer hemorrhage
4	Hyponatremia/SIADH	Hyponatremia/SIADH (narrow)	Hyponatremia; blood sodium decreased; SIADH; hypo-osmolality; water intoxication; and serum osmolality decreased
5	QTc prolongation and cardiac arrhythmias	Torsade de pointes/QT prolongation (narrow and broad)	Electrocardiogram QT prolonged; long QT syndrome; torsade de pointes; ventricular arrhythmia; sudden cardiac death; cardiac arrest; ventricular fibrillation; syncope
6	Suicidal ideation and behavior	Suicide/self-injury (broad)	Suicidal ideation; suicide attempt; completed suicide; suicidal behavior; self-injurious ideation; self-injurious behavior; intentional self-injury; suicidal depression
7	Withdrawal/discontinuation Syndrome	Drug withdrawal (broad)	Drug withdrawal syndrome; antidepressant discontinuation syndrome; dizziness; paresthesia; electric shock sensation; headache; nausea; anxiety; insomnia; irritability; agitation; sensory disturbance; tremor; confusion
8	Akathisia	-	Akathisia; restlessness; psychomotor hyperactivity; restless legs syndrome; inability to sit
9	Bleeding events (general)	Hemorrhage terms (excluding laboratory terms) (broad)	Hemorrhage; intracranial hemorrhage; cerebral hemorrhage; subarachnoid hemorrhage; subdural hematoma; epistaxis; hematoma; contusion; purpura; petechiae; ecchymosis
10	Hepatotoxicity	Drug-related hepatic disorders-comprehensive search (broad)	Hepatotoxicity; hepatic enzyme increased; ALT increased; AST increased; hepatic failure; hepatitis; liver disorder; jaundice; hyperbilirubinemia
11	Neuropsychiatric effects	-	Emotional blunting; apathy; affective flattening; cognitive disorder; memory impairment; disturbance in attention; confusional state; mental impairment; thinking abnormal; concentration impaired
12	Sleep disturbances	-	Insomnia; somnolence; hypersomnia; sleep disorder; middle insomnia; initial insomnia; terminal insomnia; abnormal dreams; nightmares
13	Gastrointestinal effects (non-bleeding)	-	Nausea; diarrhea; abdominal pain; vomiting; constipation; dyspepsia; flatulence; dry mouth
14	Metabolic/endocrine effects	Hyperglycemia/new onset diabetes mellitus (narrow and broad)	Weight increased; weight decreased; hyperglycemia; diabetes mellitus; insulin resistance; dyslipidemia; hypercholesterolemia; hypertriglyceridemia; metabolic syndrome
15	Pregnancy and neonatal complications	Pregnancy and neonatal topics (broad)	PPHN; neonatal adaptation syndrome; cardiac malformation; congenital cardiac disorder; neonatal respiratory distress; poor neonatal adaptation; premature baby; neonatal withdrawal syndrome
16	Movement disorders	-	Extrapyramidal disorder; dystonia; tremor; myoclonus; bruxism; parkinsonism
17	Falls and fractures (elderly)	-	Fall; fracture; hip fracture; osteoporosis; bone density decreased
18	Cardiovascular effects (non-arrhythmic)	Cardiomyopathy (broad)	Valvular heart disease; pulmonary hypertension; cardiac valve disorder; mitral valve incompetence; aortic valve disorder; endocardial fibrosis
19	Anticholinergic effects	-	Dry mouth; constipation; urinary retention; vision blurred; mydriasis; accommodation disorder
20	Allergic/hypersensitivity Reactions	Hypersensitivity (narrow and broad)	Hypersensitivity; angioedema; urticaria; rash; pruritus; drug hypersensitivity; anaphylactic reaction

Appendix B

**Table 6:** Key SMQs for SSRI safety monitoring SMQ: standardized MedDRA query; SSRIs: selective serotonin reuptake inhibitors; SIADH: syndrome of inappropriate antidiuretic hormone; GI: gastrointestinal

S.no	SMQ name	SMQ Code
1	Serotonin syndrome	20000208
2	Torsade de pointes/QT prolongation	20000001
3	Suicide/self-injury	20000022
4	Hyponatremia/SIADH	20000225
5	GI hemorrhage	20000269
6	Hemorrhage terms (excluding laboratory terms)	20000182
7	Drug-related hepatic disorders-comprehensive search	20000015
8	Sexual function disorders	20000212
9	Pregnancy and neonatal topics	20000181
10	Hyperglycemia/new onset diabetes mellitus	20000209
